# Hypoxic Microenvironment and Metastatic Bone Disease

**DOI:** 10.3390/ijms19113523

**Published:** 2018-11-09

**Authors:** Toru Hiraga

**Affiliations:** Department of Histology and Cell Biology, Matsumoto Dental University, 1780 Gobara-Hirooka, Shiojiri, Nagano 399-0781, Japan; bone.mets@gmail.com; Tel.: +81-263-51-2225

**Keywords:** hypoxia, hypoxia-inducible factors, bone metastasis

## Abstract

Hypoxia is a common feature of solid tumors and is associated with an increased risk of metastasis and a poor prognosis. Recent imaging techniques revealed that bone marrow contains a quite hypoxic microenvironment. Low oxygen levels activate hypoxia signaling pathways such as hypoxia-inducible factors, which play critical roles in the key stages of metastatic dissemination including angiogenesis, epithelial-mesenchymal transition, invasion, maintenance of cancer stem cells, tumor cell dormancy, release of extracellular vesicles, and generation of pre-metastatic niches. Hypoxia also affects bone cells, such as osteoblasts and osteoclasts, and immune cells, which also act to support the development and progression of bone metastases. Paradoxically, hypoxia and related signaling molecules are recognized as high-priority therapeutic targets and many candidate drugs are currently under preclinical and clinical investigation. The present review focuses on our current knowledge of the potential roles of hypoxia in cancer metastasis to bone by considering the interaction between metastatic cancer cells and the bone microenvironment. Current therapeutic approaches targeting hypoxia are also described.

## 1. Introduction

Bone is one of the most common sites of cancer metastasis [1,2]. The majority of patients (around 70%) with breast and prostate cancers and a substantial proportion of patients (30–40%) with lung cancer eventually develop bone metastases [2]. Although bone metastases, per se, are rarely responsible for cancer mortality, they are associated with significant morbidity by causing so-called skeletal-related events (SREs), including hypercalcemia, pathological fractures, spinal compression, and bone pain, indirectly leading to poor prognoses [2]. Therefore, the control of bone metastasis is a critical component in cancer treatment. However, the mechanisms of bone metastasis have not been fully elucidated.

In addition to genetic and epigenetic events, the microenvironment impacts cancer progression and metastasis through a variety of mechanisms. Hypoxia, a condition of low oxygen tension, is a hallmark of tumor microenvironments, which is caused by an imbalance between oxygen delivery and consumption [3]. Intratumoral hypoxia is associated with a significantly increased risk of metastasis and poor prognosis in cancer patients [4].

Several lines of evidence suggest that transcription factors hypoxia-inducible factors (HIFs), mediate the primary response to hypoxia [4,5]. HIFs are composed of an oxygen-sensitive α subunit (HIF-α) and a stable β subunit (HIF-1β). In mammals, three genes that encode HIF-α subunit proteins have been identified. HIF-1α and HIF-2α are broadly expressed in many human cancers, whereas little is known about HIF-3α [4,6]. Under normoxic conditions, HIF-α subunits are continuously degraded by the ubiquitin-proteasome pathway. In contrast, under hypoxic conditions, HIF-α subunits are stabilized, translocate to the nucleus, dimerize with HIF-1β, and initiate gene transcription by binding to hypoxia response elements (HREs). In addition to hypoxia, HIF-α subunits were shown to be regulated by several non-hypoxic stimuli involving kinases and reactive oxygen species [7].

In this article, the most recent understanding of the role of a hypoxic microenvironment in the development and progression of bone metastases is reviewed and discussed.

## 2. Basic Understanding of the Pathophysiology of Bone Metastasis

Once cancer cells metastasize to bone, they disrupt the physiological bone metabolism maintained by osteoblasts and osteoclasts and establish a new cellular environment more favorable for the progression of bone metastasis, the so-called “vicious cycle” [1]. In brief, osteoclast stimulating factors, such as parathyroid hormone-related protein (PTHrP), secreted by metastatic cancer cells activate bone destruction, leading to an increase in the release of bone-derived growth factors, including insulin-like growth factors (IGFs) and transforming growth factor β (TGFβ), into the bone microenvironment. These factors then act on cancer cells, thereby causing more aggressive cancer phenotypes and further bone destruction. This theory suggests that osteoclasts play pivotal roles in the process of bone metastasis.

## 3. Hypoxia and Cancer Metastasis

**Figure 1:**
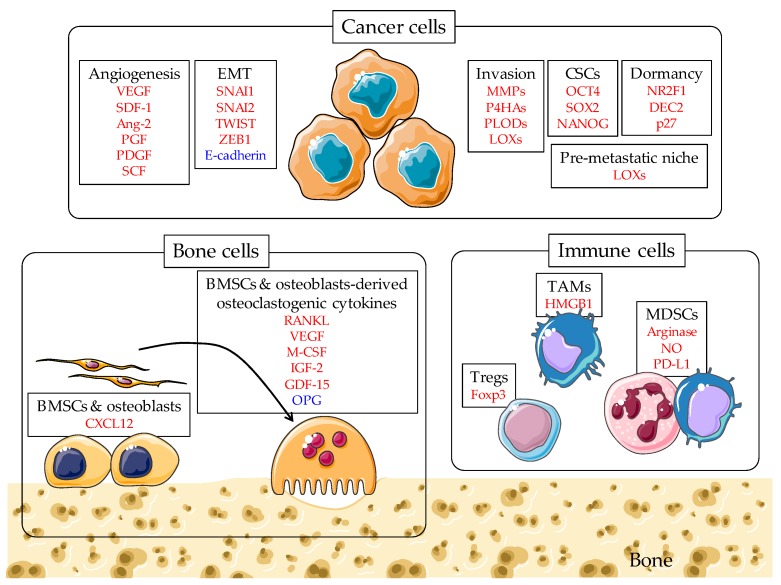
Hypoxic signaling in the bone-metastatic cascade. The figure summarizes hypoxia-induced and hypoxia inducible factor (HIF)-target genes involved in the key stages of the bone-metastatic cascade. Upregulated genes are shown in red and downregulated genes are in blue. Images adapted from Servier Medical Art (https://smart.servier.com/).

Accumulating evidence indicates that hypoxia enhances malignant phenotypes of cancer cells through enhancing angiogenesis, epithelial–mesenchymal transition (EMT), invasion, cancer stem-like phenotypes, tumor cell dormancy, release of extracellular vesicles (EVs), and generation of pre-metastatic niches, most of which are HIFs-dependent and all of which are positively correlated with cancer metastasis (Figure 1) [5,8].

### 3.1. Angiogenesis

Because one of the reasons for hypoxia caused in solid tumors is compromised blood flow, angiogenesis is an adaptive response, which stimulates O_2_ delivery and supports tumor growth. HIFs function as master regulators of angiogenesis by upregulating the expression of genes involved in angiogenic responses, such as vascular endothelial growth factor (VEGF), stromal-derived factor-1 (SDF-1), angiopoietin-2 (Ang-2), placental growth factor (PGF), platelet-derived growth factor (PDGF), and stem cell factor (SCF) [5]. It has been well-described that angiogenesis is essential for cancer metastasis [9]. There have also been several reports showing the relationship between angiogenesis and bone metastasis. A preclinical study showed that the angiogenesis inhibitor TNP-470 suppressed bone metastases of MDA-MB-231 human breast cancer cells [10]. The study, using clinical samples of breast cancer, revealed that the microvessel density in the primary tumors had a strong correlation with the occurrence of bone metastases [11].

### 3.2. EMT

EMT is the transition of epithelial cells to cells with mesenchymal phenotypes [12]. It has been demonstrated that HIF-1 downregulates E-cadherin, a specific marker for epithelial cells, by enhancing the expression of so-called EMT-activating transcription factors, including *SNAI1*, *SNAI2*, *TWIST*, and *ZEB1* [5,8]. Although the relationship with hypoxia and HIFs was not determined, the overexpression of *TWIST1* was shown to facilitate the development of bone metastases of MDA-MB-231/B02 human breast cancer cells in mice through a mechanism dependent on microRNA-10b (miR-10b) [13]. Liu et al. reported that the expression of ZEB1 in bone-metastatic small cell lung cancer (SCLC) tissues and cell lines was higher than that in non-metastatic ones [14]. They also demonstrated that *ZEB1* knockdown and *ZEB1* overexpression inhibited and promoted bone metastases of SBC-3 and SBC-5 human SCLC cells in mice, respectively [14].

### 3.3. Invasion

The local invasion of tumor cells from the primary tumors to the adjacent stroma is a first step in the metastasis cascade. The degradation of extracellular matrix (ECM) is one of the mechanisms that tumor cells utilize to accelerate the invasion. Several proteinases are involved in this process. Among these enzymes, the expression of matrix metalloproteinases (MMPs), including MMP2, MMP9 and MMP14, are regulated by HIFs [5]. HIFs also modulate ECM remodeling through increased expression of prolyl-4-hydroxylases (P4HA1 and P4HA2), procollagen-lysine,2-oxyglutarate 5-dioxygenases (PLOD1 and PLOD2), and lysyl oxidases (LOX, LOXL2, and LOXL4), which are required for cancer cell invasion [5].

### 3.4. Cancer Stem Cells (CSCs)

The cancer stem cell (CSC) hypothesis proposes that only a small fraction of tumor cells have tumor-initiating potential and are able to self-renew and differentiate into the heterogeneous cell populations that compose tumors [15]. These stem-like properties are required to initiate secondary tumor formation in distant organs. Our preclinical studies demonstrated that cancer stem-like phenotypes contribute to the development of bone metastases of human breast cancer MDA-MB-231 cells [16] and mouse breast cancer 4T1 and Jyg-MC(A) cells [17] in mice.

Hypoxia was shown to induce the cancer stem-like phenotypes in an HIF-dependent manner firstly in glioma [18] and, subsequently, in several types of cancer, including breast cancer [19]. Hypoxia and HIFs promote the generation and maintenance of CSCs through the expression of genes, including octamer-binding transcription factor 4 (*OCT4*), sex determining region Y-box 2 (*SOX2*), and *NANOG* [20].

### 3.5. Dormancy

Tumor dormancy is defined as a temporary mitotic and growth arrest, which might explain local recurrences and metastases at different time points after treatment [21]. Metastases originate from disseminated tumor cells (DTCs), which often undergo a period of dormancy [22]. Bone marrow is one of the most frequent sites in which DTCs are detected [21]. In this case, bone can be a target organ of metastasis, but it might also serve as a transit site from which cells can again disseminate to their final metastatic organs.

It has been suggested that tumor cell dormancy is regulated by hypoxia. Fluegen et al. showed that hypoxic microenvironments upregulate key dormancy genes, such as *NR2F1*, *DEC2*, and *p27*, in head and neck and breast cancer cells in mice and humans, which was accompanied by the overexpression of HIF-1 and glucose transporter 1 (GLUT1) [23]. Furthermore, post-hypoxic DTCs become quiescent in metastatic sites and evade chemotherapy. Another group reported that several cycles of hypoxia and re-oxygenation generate a hypoxia-resistant cell line, which can survive under hypoxic conditions by entering into a dormant state that is reversible upon normoxia exposure [24]. In contrast, Johnson et al. proposed the opposite effects of hypoxia on tumor cell dormancy in bone marrow [25]. They revealed that leukemia inhibitory factor receptor (LIFR)-signal transducer and activator 3 (STAT3)-suppressor of cytokine signaling 3 (SOCS3) signaling in DTCs confers a dormancy phenotype in response to LIF, a member of the IL-6 family of cytokines and produced by osteoblast-lineage and bone marrow stromal cells. The pathway is inhibited by hypoxia and HIF signaling, leading to the transition from a dormant to an invasive phenotype. Further studies are required to clarify the complex relationships between hypoxia and tumor cell dormancy.

### 3.6. Extracellular Vesicles (EVs)

EVs, including exosomes (30–100 nm diameter) and microvesicles (100–1000 nm diameter), are cell-derived vesicles, which are now recognized as major players in cell–cell communication [26]. Cancer-associated EVs also promote angiogenesis, CSCs, EMT, and metastasis through their content, such as various types of proteins, RNAs, and DNAs. EVs derived from prostate cancer (PC3), myeloma (U266, MM1S, and OPM2), and lung cancer (CRL-2868) cells were shown to stimulate osteoclast formation [27,28,29], whereas those from prostate cancer (TRAMP-C1) cells inhibited osteoclast formation [30]. Exosomes derived from bone marrow stromal cells (BMSCs) in multiple myeloma facilitated tumor growth, which was inhibited by exosomes from normal BMSCs [31]. Another report revealed that exosomes from BMSCs promoted dormancy in metastatic breast cancer cells by exosomal transfer of miRs [32]. Furthermore, Hashimoto et al. showed that exosomes from prostate cancer cells containing hsa-miR-940 induced bone metastases with an osteoblastic phenotype [33]. These findings imply the presence of exosome-mediated complex cell–cell communication in the bone metastatic microenvironment.

It has been reported that hypoxia increases the release of EVs from cancer cells [34]; however, the contribution to metastatic bone diseases remains to be investigated.

### 3.7. Pre-Metastatic Niche

The pre-metastatic niche, a favorable microenvironment created in secondary organs for subsequent metastases, is formed mainly by primary tumor-derived factors, tumor-mobilized bone marrow-derived cells (BMDCs), and local stromal components [35]. Recent studies revealed that the *LOX* family members, including *LOX*, *LOXL2*, and *LOXL4*, are key HIF-1-regulated genes involved in BMDC recruitment to promote pre-metastatic niche formation [36]. *LOX* has also been shown to play a role in the formation of pre-metastatic bone lesions, which will be described in detail in Section 6.

## 4. Hypoxia and Bone Cells

According to a study in which partial pressure of oxygen (pO_2_) in the calvariae of live mice was measured using two-photon phosphorescence lifetime microscopy, the absolute pO_2_ in bone marrow was <32 mmHg (4.2%) despite the high vascular density [37]. The heterogeneous pO_2_ in bone marrow was lowest in deeper perisinusoidal regions (9.9 mmHg, 1.3%). These data indicate that bone is a quite hypoxic microenvironment.

### 4.1. Effects of Hypoxia on Osteoclasts

Hypoxia was shown to increase osteoclast formation in vitro [38,39], which was supported by findings that hypoxia stimulates the production of pro-osteoclastogenic cytokines, such as receptor activator of nuclear factor-κB ligand (RANKL), VEGF, macrophage colony-stimulating factor (M-CSF), IGF-2, and growth differentiation factor-15 (GDF-15), and inhibits the production of osteoprotegerin (OPG), an inhibitor of osteoclast differentiation [40]. Furthermore, the report by Miyauchi et al. suggested that hypoxia-induced osteoclast differentiation is mediated, at least in part, by HIF-1α [41]. They further proposed that HIF-1α is required for osteoclast activation [41]. Several studies, including ours, demonstrated that acidosis caused by hypoxia also promoted osteoclast formation and activity, which was mediated by the up-regulation of RANKL and nuclear factor of activated T cells cytoplasmic 1 (NFATc1) [39,42]. Hypoxia most likely acts to enhance osteoclastic bone destruction and bone metastasis (Figure 1).

### 4.2. Effects of Hypoxia on Osteoblasts

In vitro studies showed that hypoxia exhibited inhibitory effects on the differentiation and bone-forming capacity of osteoblasts [39,43]. In contrast, mice with osteoblast-specific deletion of von Hippel-Lindau (VHL) and osteoblast-specific overexpression of HIF-1α exhibited increased bone volume and osteoblast numbers [44,45], suggesting that hypoxia and HIF-1α activation have positive effects on bone formation in vivo. Further studies are required to explain the discrepancy between in vitro and in vivo data.

A recent study by Devignes et al. demonstrated that activated HIF signaling in osteoblast-lineage cells promotes bone metastases in mice [46]. Mechanistically, they found that activation of HIF signaling in osteoprogenitor cells increased the production of C-X-C motif chemokine ligand 12 (CXCL12), which leads to an increase in proliferation and dissemination of breast cancer cells through direct activation of C-X-C motif chemokine receptor 4 (CXCR4), a receptor for CXCL12. Of note, these effects were also found in organs other than bone, such as lung, suggesting that HIF signaling in osteoblast-lineage cells might cause tumorigenesis beyond the local bone microenvironment (Figure 1).

## 5. Hypoxia and Immune Cells in Bone

Bone is a unique organ, which belongs not only to the musculoskeletal system but also to the immune system [47,48]. In bone, multiple types of immune cells and their progenitor cells share the same microenvironment with bone cells. Recent emerging evidence suggests the mutual relationship between bone and immune cells in their differentiation and function. Since 2000, the term “osteoimmunology” has been used to describe this discipline [49]. Thus, as well as cancer cells and bone cells, immune cells are one of the major players in metastatic bone lesions.

Malignant tumor cells have many mechanisms to make their microenvironment immunosuppressive, including the ability to suppress immune responses through immune check points, such as the cytotoxic T-lymphocyte antigen-4 (CTLA-4) and programmed cell death protein 1 (PD1) pathways [50]. In addition, hypoxia has recently been implicated as an important factor driving the immunosuppressive tumor microenvironment, which is created, at least in part, by the infiltration of immunosuppressive cells, such as tumor-associated macrophages (TAMs), myeloid-derived suppressor cells (MDSCs), and regulatory T (Treg) cells [51]. However, little is known about the involvement of these immune cells in bone metastasis (Figure 1).

### 5.1. TAMs

TAMs are macrophages infiltrating the tumor microenvironment. It has been demonstrated that the density of TAMs correlates with poor prognosis in several types of cancer [52]. TAMs can be divided into classically activated M1-like and alternatively activated M2-like subtypes [53]. M1-like TAMs are known to exert anti-tumor activity by promoting proinflammatory effects and immune responses. On the other hand, M2-like TAMs enhance tumor angiogenesis, matrix remodeling, tumor cell migration and invasion, and immunosuppression, resulting in tumor progression. TAMs isolated from established metastatic tumors are predominantly polarized to an M2-like phenotypes [53]. Several mediators, including interleukin-4 (IL-4) and IL-10, are involved in M2-like polarization. Huber et al. reported that hypoxia induced the secretion of high mobility group box-1 (HMGB1) from melanoma cells, which caused IL-10 production and M2-like TAM accumulation within the tumor [54], suggesting that hypoxia contributes to the conversion of TAMs to an immunosuppressive M2-like phenotype. Laoui et al. revealed that M2-like TAMs are preferentially attracted to hypoxic regions [55]. Furthermore, tumor-derived lactic acid, whose levels are elevated by hypoxic metabolism, promotes M2-like polarization in a HIF-1α-dependent manner [56]. These findings suggest that hypoxia is associated with the accumulation of M2-like TAMs and enhances immunosuppressive microenvironment. In addition, hypoxia is also shown to augment macrophage-mediated T-cell suppression in a manner dependent on HIF-1α [57].

### 5.2. MDSCs

MDSCs are also tumor-infiltrating myeloid cells which impair immune responses through various mechanisms [58]. HIF-1α has been reported to regulate the function and differentiation of MDSCs within the hypoxic tumor microenvironment [59]. According to Corzo et al., tumor-derived MDSCs possess higher immunosuppressive capacity than splenic MDSCs, which is mainly due to the HIF-1α-dependent induction of arginase activity and nitric oxide (NO) production [59]. Furthermore, Noman et al. showed that hypoxia upregulates the expression of programmed death-ligand 1 (PD-L1) in MDSCs and increases MDSC-mediated T cell tolerance [60].

### 5.3. Tregs

Tregs are a highly immunosuppressive fraction of CD4^+^ cells [61]. In tumors, Tregs exhibit activated phenotypes, including high expression of immune suppression-related molecules such as CTLA-4. Several studies reported that hypoxia is associated with the selective accumulation of Tregs in tumors, which not only suppresses antitumor responses but also promotes angiogenesis [62]. As a mechanism, hypoxia was shown to potently increase the expression of forkhead box P3 (Foxp3), a master regulator in the development and function of Tregs [63].

## 6. Hypoxia, HIFs, and Bone Metastasis

### 6.1. Hypoxia and Bone Metastasis

Intratumoral hypoxia is a common feature of cancers. According to Vaupel et al., overall median pO_2_ in cancers, including uterine cervix, head and neck, and breast cancers, was 10 mm Hg, which was markedly lower than that in normal tissues, which ranges from 24 to 57 mm Hg [64]. Although the absolute values of pO_2_ in bone metastases in cancer patients are still unknown, our mouse study revealed the presence of pimonidazole-positive hypoxic regions in the bone metastases of MDA-MB-231 cells in nude mice [39]. Because pimonidazole accumulates in cells that have pO_2_ less than 10 mmHg (1.3%) [65], the result suggests that the hypoxic levels in bone metastases in this animal model are equivalent to those in human cancers [64].

As described in Section 3, hypoxia is closely associated with malignant behaviors of cancer cells. Consistent with this notion, our previous study demonstrated that a hypoxia-targeted agent, trans-activator of transcription (TAT)-oxygen-dependent degradation domain (ODD)-procaspase-3 (TOP3), whose details will be described in Section 7, significantly reduced bone metastases of MDA-MB-231 cells in mice [39]. Furthermore, as mentioned in Section 4, hypoxia and the resulting metabolic acidosis stimulate the differentiation of osteoclasts, key players in the vicious cycle of bone metastasis [1]. These data suggest the promoting roles of hypoxia in bone metastases; however, our study did not fully address whether or how hypoxia in primary tumors controls spontaneous metastasis to bone.

### 6.2. HIFs and Bone Metastasis

Gene expression analysis with cDNA microarrays showed that the expression of HIF-1α was three-fold higher in primary tumors of breast cancer patients with bone micrometastases than in those without the micrometastases, which was associated with the down-regulation of genes responsible for HIF-1α degradation, such as *VHL* and cullin-2 [66]. The results suggest that HIF-1α may promote tumor dissemination to bone.

In our preclinical model, an immunohistochemical study confirmed the nuclear HIF-1α expression in tumor cells in the bone metastases of MDA-MB-231 cells in nude mice, which co-localized or surrounded the pimonidazole-positive cells [39]. Furthermore, the stable expression of constitutive-active HIF-1α increased and dominant-negative HIF-1α decreased the development of bone metastases [39]. The results were confirmed by Lu et al.; although the effects were not specific to metastases to bone [67]. They also identified two genes, dual specificity protein phosphatase 1 (*DUSP1*) and *CXCR4*, as hypoxia-activated bone metastasis-associated genes.

PTHrP is a potent stimulator of osteoclastic bone destruction and bone metastasis [68]. Manisterski et al. revealed that hypoxia enhanced the expression of PTHrP in cancer cells mediated through HIF-2α but not HIF-1α [69]. It is well-known that PTHrP expression is also induced by TGFβ, one of the growth factors abundantly stored in bone matrix and the key molecule in the vicious cycle of bone metastasis [1]. Dunn et al. reported that hypoxia and TGFβ regulate a common set of tumor genes, including PTHrP [70]. Furthermore, because inhibition of either pathway decreased bone metastases, with no further effect of double blockade, they proposed that hypoxia and TGFβ signaling drives bone metastases in parallel. Collectively, these results suggest the critical role of HIFs in bone metastases.

### 6.3. Emerging Roles of Lysyl Oxidase (LOX) in Bone Metastasis

LOX is a secreted copper-dependent amine oxidase, whose primary function is the covalent crosslinking of collagens and elastins in the ECM [71]. However, recent studies demonstrated that LOX also plays critical roles in hypoxia-induced cancer metastasis. Erler et al. firstly described that LOX expression is regulated by HIF-1 and the inhibition of LOX eliminates metastasis in mice [72]. Mechanistically, LOX increases cell motility through up-regulating focal adhesion kinase activity and cell-matrix adhesion. It was also revealed that LOX secreted by hypoxic cancer cells acts to form a pre-metastatic niche by recruiting CD11b^+^ myeloid cells [73].

It has also been reported that LOX plays critical roles in cancer cell colonization in bone. According to Cox et al., hypoxia is specifically associated with bone relapse in patients with estrogen receptor (ER)-negative breast cancer [74]. Their study using spontaneously metastasizing ER^-^ breast cancer 4T1 cells, which express high levels of LOX, demonstrated that inhibition of LOX by short hairpin RNA or anti-LOX antibody abrogates bone metastasis formation. Conversely, experiments using SW480, a non-metastatic colon cancer cell line with low LOX expression, showed that overexpression or systemic delivery of LOX leads to bone metastasis formation. These findings are further supported by the report by Reynaud et al.; however, the effects were independent of HIF-1 but dependent on LOX-driven IL-6 production [75]. They also revealed that LOX stimulates osteoclastogenesis and inhibits osteoblastogenesis [74,75]. Thus, it is most likely that LOX-induced disrupted bone homeostasis leads to the formation of focal pre-metastatic lesions in bone. Additionally, LOX is expressed in osteoblasts, which is induced by TGFβ released during bone resorption [76]. The findings fit naturally into the vicious cycle theory. These results collectively suggest the critical contribution of LOX to bone metastases. Thus, LOX could be a novel target for therapeutic intervention [77].

It is of interest that an in vitro study by Cox et al. showed that LOX stimulated osteoclast formation independently of RANKL, which was mediated through the direct action on NFATc1, the master regulator of osteoclastogenesis [74]; however, later studies failed to reproduce these findings [75,78]. There is a clear need for further study of the precise molecular mechanisms underlying LOX-driven osteoclastogenesis.

## 7. Target Hypoxia

Considering the critical contributions as described above, hypoxia and HIFs are promising therapeutic targets in treating bone metastases. One approach utilizes bioreductive or hypoxia-activated prodrugs (HAPs) to eradicate hypoxic cells [79,80]. Another strategy is the inhibition of HIFs and their downstream targets or upstream signaling molecules.

### 7.1. HAPs

HAPs are activated by enzymatic reduction at low oxygen tension. The majority of HAP metabolites result in DNA damage by interfering with DNA replication. Several HAPs, including AQ4N, PR-104, and TH-302 (evofosfamide), are currently undergoing clinical trials. Some other compounds, such as TOP3, are also under development. However, to date, there are only a few studies available that have investigated the effects on metastatic bone diseases.

#### 7.1.1. AQ4N

AQ4N is enzymatically converted to a cytotoxic DNA-binding agent, AQ4, under hypoxic conditions. AQ4 non-covalently binds to DNA to facilitate anti-tumor activity and selectively inhibits the activity of the DNA separation enzyme topoisomerase II [81]. A phase I clinical trial in patients with several types of solid tumors demonstrated that AQ4N was well-tolerated and activated selectively in hypoxic regions in solid tumors [82,83].

#### 7.1.2. PR-104

PR-104 is a phosphate ester that is hydrolyzed and reduced under hypoxic conditions to release amine (PR-104M) and hydroxyl-amine (PR-104H) nitrogen mustards [80]. Subsequently, these reactive nitrogen mustards crosslink DNA and cause cytotoxicity in cancer cells. According to a phase I/II study in patients with acute myeloid leukemia (AML) and acute lymphoblastic leukemia (ALL), 32% of AML patients and 20% of ALL patients showed response to treatment with PR-104 (ClinicalTrials.gov NCT01037556) [84].

#### 7.1.3. TH-302 (evofosfamide)

TH-302 is a 2-nitroimidazole prodrug that selectively releases bromo-isophosphoramide which crosslinks DNA under hypoxic conditions [80]. A preclinical study showed that in vivo treatment of the 5T33MM mouse model of multiple myeloma with TH-302 induced apoptosis of tumor cells within the bone microenvironment [85]. Furthermore, Liapis et al. demonstrated that TH-302 significantly delayed the growth of human breast cancer MDA-MB-231-TXSA cells in mouse tibiae [86]. The combination with paclitaxel exhibited greater inhibitory effects. However, phase III studies in advanced unresectable or metastatic pancreatic adenocarcinoma (ClinicalTrials.gov NCT01746979) and in soft tissue sarcoma (ClinicalTrials.gov NCT01440088) reported that TH-302 did not achieve primary overall survival endpoints in combination with doxorubicin or gemcitabine [87].

#### 7.1.4. TOP3

TOP3 is a fusion protein that comprises of the protein transduction domain embedded in the human immunodeficiency virus (HIV)-TAT protein, ODD, and procaspase-3 [88]. TOP3 is degraded under normoxic conditions; however, under hypoxic conditions, the protein is stabilized and causes caspase-3-induced cell death. Furthermore, because HIV-TAT fusion proteins freely diffuse through the cell membrane, TOP3 can be delivered to tissues with compromised blood supply [89]. We reported previously that TOP3 selectively induced apoptosis in hypoxic tumor cells in vitro and significantly reduced bone metastases of MDA-MB-231 cells in mice [39].

### 7.2. Targeting HIFs

Although targeting HIFs themselves remains challenging, several clinical trials using chemotherapeutic agents, such as CRLX101 (ClinicalTrials.gov NCT02187302) [90], letrozole [91], bortezomib (ClinicalTrials.gov NCT00428545) [92], EZN-2208 (ClinicalTrials.gov NCT01251926), temsirolimus (ClinicalTrials.gov NCT00761644) [92], and topotencan (ClinicalTrials.gov NCT00436644) [93], which are expected to reduce the expression and/or transcriptional activity of HIFs, have been conducted. Some of them showed favorable results.

As an approach to directly target HIFs, miRs have been investigated at preclinical levels [94]. Several miRs are induced in response to low oxygen, the majority of which are also overexpressed in a variety of human tumors. Some of those, such as miR-26, -107, and -210, were shown to interfere with pro-apoptotic signaling under a hypoxic environment, suggesting a possible involvement of these miRs in tumor formation [95]. Zhou et al. reported that miR-33b targeted HIF-1α and inhibited the proliferation and migration of osteosarcoma cells [96].

## 8. Conclusions

As well as tumor tissues, recent advances in imaging technology confirmed that bone is a generally hypoxic microenvironment. The studies described above collectively suggest the critical roles of hypoxia and related genes in promoting cancer metastasis; however, the specific contribution to bone metastasis remains less clear. Continued studies to further understand specific mechanisms of the roles of hypoxia in bone metastasis are clearly warranted and may likely lead to new and innovative therapeutic strategies to block and cure bone metastasis.

## Abbreviations

ALL	acute lymphoblastic leukemia
AML	acute myeloid leukemia
Ang	angiopoietin
BMDCs	bone marrow-derived cells
BMSCs	bone marrow stromal cells
CSCs	cancer stem cells
CTLA-4	cytotoxic T-lymphocyte antigen-4
CXCL12	C-X-C motif chemokine ligand 12
CXCR4	C-X-C motif chemokine receptor 4
DTCs	disseminated tumor cells
DUSP1	dual specificity protein phosphatase 1
ECM	extracellular matrix
EMT	epithelial-mesenchymal transition
ER	estrogen receptor
EVs	extracellular vesicles
Foxp3	forkhead box P3
GDF	growth differentiation factor
GLUT1	glucose transporter 1
HAPs	hypoxia-activated prodrugs
HIFs	hypoxia-inducible factors
HIV	human immunodeficiency virus
HMGB1	high mobility group box-1
HRE	hypoxia response element
IGFs	insulin-like growth factors
IL	interleukin
LIFR	leukemia inhibitory factor receptor
LOX	lysyl oxidase
M-CSF	macrophage colony-stimulating factor
MDSCs	myeloid-derived suppressor cells
miR	microRNA
MMPs	matrix metalloproteinases
NFATc1	nuclear factor of activated T cells cytoplasmic 1
NO	nitric oxide
OCT4	octamer-binding transcription factor 4
ODD	oxygen-dependent degradation domain
OPG	osteoprotegerin
P4HA	prolyl-4-hydroxylase
PD1	programmed cell death protein 1
PDGF	platelet-derived growth factor
PD-L1	programmed death-ligand 1
PGF	placental growth factor
PLOD	procollagen-lysine, 2-oxyglutarate 5-dioxygenase
pO2	partial pressure of oxygen
PTHrP	parathyroid hormone-related protein
RANKL	receptor activator of nuclear factor-κB ligand
SCF	stem cell factor
SCLC	small cell lung cancer
SDF-1	stromal-derived fator-1
SOCS3	suppressor of cytokine signaling 3
SOX2	sex determining region Y-box 2
SREs	skeletal-related events
STAT3	signal transducer and activator 3
TAMs	tumor-associated macrophages
TAT	trans-activator of transcription
TGFβ	transforming growth factor β
TOP3	TAT-ODD-procaspase-3
VEGF	vascular endothelial growth factor
VHL	von Hippel-Lindau
LD	linear dichroism
